# How to Enhance the Motivation for Drug Detoxification: Consciousness Guidance and Behaviour Restriction of Family Intergenerational Ethics

**DOI:** 10.3390/ijerph19010366

**Published:** 2021-12-30

**Authors:** Pei Hong, Shengnan Li, Yanping Yu, Quanyang Deng

**Affiliations:** 1 Department of Social Work, Central China Normal University, Wuhan 430079, China; hongpei@ccnu.edu.cn (P.H.); lsn2021@mails.ccnu.edu.cn (S.L.); 2 Department of Sociology, Wuhan University of Technology, Wuhan 430070, China; 3 School of Public Administration, Nanchang University, Nanchang 330031, China; quanyangdeng@163.com

**Keywords:** motivation for drug rehabilitation, family ethics, intergenerational responsibility

## Abstract

Assisting substance users to recover from the behaviour of drug addiction and maintain long-term rehabilitation is a long and complicated process, in which the motivation to undergo drug rehabilitation plays a decisive role. So far, the cultural connotation of family and its mechanism of promoting behavioural change of substance users have not been fully explored. Through in-depth interviews with 15 drug rehabilitants, among which there were 7 women and 8 men, it is found that the motivation for drug rehabilitation is stimulated under the guidance and restriction of family ethics based on obligation and responsibility, which is mainly reflected in the longitudinal intergenerational responsibility. On the one hand, negative consequences such as intergenerational liability deficit and reputation damage lead substance users to reflect on ethical values. On the other hand, disciplines such as intergenerational responsibility and obligation and mutual assistance can correct the actual behaviour of substance users in ethical practice. In contrast to Western countries, which focus on external environmental factors such as family function, family relationships and family support, the motivation for drug rehabilitation in China places more emphasis on their identity and role as family members and corresponding responsibilities, which provides inspiration for developing social work services for substance users from family cultural norms.

## 1. Introduction

**Figure 1:**
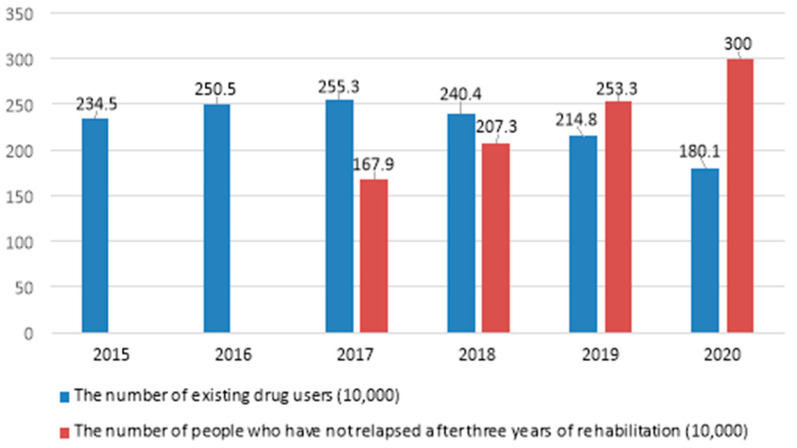
The number of existing drug users and the number of rehabilitants without relapse for three years in China (2015–2020). (Sources: The data are derived from the China Drug Situation Report issued by the Office of the National Anti-Drug Committee of China between 2015 and 2020. Retrieved from http://www.nncc626.com/) (accessed on 30 June 2021).

More than 130 countries and regions are faced with the problem of drug consumption, and more than 170 countries and regions are faced with the problem of drug cultivation and trafficking [1]. The number of drug users in the world currently exceeds 200 million, and the number of drug-related deaths reaches 200,000 every year [2]. Moreover, we have to face the global problem of “relapse after drug detoxification”. One clinical treatment study estimates that more than two-thirds of substance users relapse within weeks to months after rehabilitation [3]. A large number of drug users return to taking drugs after treatment; this is the most prominent problem in substance use treatment in almost all drug-infested countries and one of the major obstacles in the fight against drugs [4]. China has made substantial progress in the prevention and control of drug abuse in recent years. The number of existing substance users (excluding the number of people who have not relapsed after three years of rehabilitation, the number of deaths and the number of people who left the country) has declined for three consecutive years, and the number of people who have not relapsed after three years of rehabilitation has increased for many years, and even surpassed the existing number of substance users for two consecutive years (see Figure 1). However, the drug problem remains difficult to control. On the one hand, the number of drug users is huge. According to the Report on the Drug Situation in China in 2020, by the end of 2020, there were 1.801 million drug users in China, including 155,000 newly discovered drug users [5]. This sharp decline was influenced to some extent by the difficulty of obtaining drugs for substance users during the period of strict epidemic prevention and control. The World Drug Report 2020 released by the United Nations Office on Drugs and Crime (UNODC) also explained that the decline of substance users caused by the restrictions on actions and gatherings as well as the need to maintain social distance in order to prevent the spread of the COVID-19 epidemic is likely to end when the restrictions are lifted [6]. In addition, the actual number of drug users often far exceeds the officially announced numbers. On the other hand, the relapse rate remains high. The most common feature of drug addiction is a high rate of relapse. According to the Report on the Drug Situation in China released by the Office of the National Anti-Drug Committee over the years, about 500,000 relapsed drug abusers are arrested every year. Drug abuse problems caused by relapse behaviour continue to recur, which not only causes great harm and loss to drug users themselves and their families [7], but it may also induce a series of illegal and criminal activities such as theft, robbery and fraud [8], bringing potential risks and dangers to public security.

Lack of motivation is considered to be one of the most common causes of relapse [9]. In view of the key role of detoxification motivation in the change of addictive behaviour, researchers have accumulated much evidence to prove the relationship between detoxification motivation and rehabilitation [10], and how to enhance the motivation of substance users [11]. These studies indicate that the motivation for detoxification is a multidimensional and multilevel complex phenomenon, which is affected by multiple factors [12]. Among them, as a micro system that has a direct influence on individual behaviour [13], the important role of family in stimulating substance users to enhance motivation for change [14] has aroused extensive attention. Numerous studies support the view that family factors play a positive role in enhancing the motivation for drug detoxification. However, most of the relevant research is quantitative [15,16] and there is a lack of further understanding and interpretation of the mechanism of family factors. At the same time, it is obviously different from Western countries in terms of value pursuit and ethical orientation in Chinese society [17]. Family is not only an individual’s cultural unit, but also an important carrier to obtain code of conduct. As a result, the impact of family on individuals and the way it operates vary from culture to culture. However, little attention has been given to the impact of family factors on the substance users’ motivation for drug detoxification in Eastern societies. How family ethical norms and responsibilities, which have a profound influence on the behavioural logic of Chinese people, promote the drug detoxification motivation of substance users has not been fully explored.

To address the lacuna in knowledge, based on a qualitative study in China, the present study explores how family factors, particularly family ethics, facilitate substance users’ motivation to put an end to their drug use.

## 2. Literature Review

### 2.1. Two Orientations on Motivation for Drug Detoxification

Recovering from the behaviour of drug addiction and maintaining long-term rehabilitation is a long and complicated process that involves many influencing factors and constraints. Many studies have shown that the motivation and willingness to stop using drugs is the starting point of the process [18], and can effectively predict the tendency to relapse [19] and help to increase the time without relapsing into addiction [20] and the success rate of rehabilitation [21,22]. To some extent, recovery from drug abuse is actually a process centred on stimulating and maintaining the motivation for change [23]. Positive behavioural changes are likely to occur only when a drug abuser reawakens cognitively [24]. Previous studies on the motivation for drug detoxification mainly focus on the investigation of motivation status and the analysis of influencing factors.

Globally, researchers usually conduct survey studies and adopt related scales to investigate the motivational status and the characteristics of rehabilitants. The scales commonly used to measure the motivation of substance users [25,26,27,28,29] are basically the specific application of the Wheel of Change model [30], which mainly focuses on the attitude, intention and behavioural characteristics of substance users, and reflects the strong and weak state of substance users’ motivation through the psychological and behavioural changes in different stages of drug rehabilitation. Chinese researchers have also developed a number of scales on motivation for drug treatment [31,32,33], which focus on different structures and types to reflect the level of motivation of substance users. Obviously, due to the limitation of the scale measurement method, the existing studies either show the detoxification motivation in different stages of the process of addiction behaviour change or statically present the scores of the detoxification motivation in different dimensions based on the perspective of the “structure factor”, and rarely consider topics of how the motivation to change could be enhanced. Even studies that use the grounded theory approach to explore why substance users put an end to their drug use [34] just simply list the important factors that contribute to their decision to “quit” and do not adequately explain why these factors or events lead to their decision. Therefore, there is still enough room for discussion on how to enhance the motivation of substance users to put an end to their drug use.

There are two different orientations in the analysis of influencing factors. The individual psychological orientation focuses on the factors related to individual psychological cognition and psychological state, which may be related to the general understanding of detoxification motivation as a kind of psychological cognition [35]. Several studies have demonstrated the negative effects of traumatic events and their negative emotional experiences on the detoxification motivation [36], and further analysed the mediating effects of self-concept [37], self-esteem and emotional regulation self-efficacy [38] between traumatic experiences and detoxification motivation. In addition, the relationship between personality characteristics and coping style [39], emotional regulation ability [40], positive psychological capital [41], self-awareness [42] and the motivation for detoxification have also been explored. The systematic orientation focuses on the influence of the social environment factors on the motivation for drug detoxification, such as social support [43,44,45,46,47]. Many research findings indicated that family environment and family support are important factors affecting the motivation and effect of drug rehabilitation among substance users [48,49]. In addition, as early as the 1970s, some researchers suggested that substance users undergoing rehabilitation should not be separated from their families [50], and it would be futile to treat substance users separately from their families if family factors were not taken into account in drug treatment [51].

### 2.2. The Important Influence of Family on the Motivation for Detoxification

A large number of studies have discussed the important role of family in the enhancement of motivation for drug rehabilitation, but there are controversies in the research conclusions; in other words, research on the relationship between family factors and detoxification motivation has not generated a consistent conclusion.

On the one hand, existing studies have emphasised or demonstrated the relationship between family function [52,53], family support [54,55], family relationships [56,57] and substance users’ involvement in drug rehabilitation treatment. It is believed that the more perfect the family system, the better the family function, the higher the degree of family support for substance users, the stronger the willingness to invest in drug treatment and the motivation to maintain a drug-free life [58]. In the aspect of “family support”, an interview study on 40 drug users in Nigeria found that different forms of support, including financial, material and spiritual support, provided by families were important catalysts for substance users to achieve rehabilitation [59]. In addition, a number of questionnaire studies have confirmed the predictive effect of family support on drug rehabilitation motivation [60], relapse tendency [61] and maintenance effect [62]. In the aspect of “family relationship”, the interaction between substance users and family members [63] and their ability to express themselves emotionally [64] could affect the relapse tendency or the success rate of drug rehabilitation. Other studies have shown that family participation and the repair of their relationship with their families are the most important predictors of drug abusers’ participation in treatment [65], and could encourage substance users to admit that they are willing to change [66] and maintain positive changes [67] and stability in terms of detoxification [68]. In the annual report issued by the United Nations Office on Drugs and Crime (UNODC), it has been repeatedly emphasised that the improvement of family support system and relationship was of great benefit for the treatment of drug addiction [69]. Currently, there are many substance use treatment programmes in the United States that include the families of substance users in the treatment scope, involve the correction and adjustment of the family environment, including solve the original problems in the family, change the interaction pattern among the family members, improve the relationship among the family members and enhance parenting competencies [70]. Family-focused intervention models have also yielded good results, with nearly half (46%) of substance users successfully came off drugs with the help of their families [71].

On the other hand, some studies also suggested that although many family factors may have a direct or indirect impact on substance users, relied solely on the positive support of the family system was not a panacea [72]. McKay et al. conducted a longitudinal study on 14 factors that have effectively assisted substance users to put an end to their drug use and maintained a drug-free life, and found that the positive support and positive social network of the family system was only a secondary curative factor, and the real main predictors were the high self-efficacy, self-help participation, readiness to change and depression of substance users [73]. The results of a questionnaire survey in Indonesia also showed that although family support had a significant positive impact on substance users’ motivation, the main indicator that had a major impact on family support was the rehabilitants’ perception of whether their family could meet their well-being needs. At the same time, individual guilt and a sense of responsibility to oneself, family, community, government and God would also motivate substance users to stop using drugs [74]. Some studies of substance users in Taiwan have the same findings, i.e., that there was no simple and objective standard for whether family factors have an adequate protection effect for substance users [75], whether or not substance users themselves have good psychological adjustment ability [76], and that how to understand and interpret family environment factors [77] was the key. The latest studies also found that psychological capital played a mediating role between family closeness and relapse tendency [78], and life strategies could also regulate the mediating effect of psychological capital [79]. In conclusion, the family can only provide limited assistance, and the important factor that really determines whether substance user to “quit drugs” or not is still the individual’s psychological state and the significance given to family factors.

Existing studies have fully demonstrated the important influence of family on the motivation of detoxification, but mainly discussed whether some family factors would affect the enhancement of motivation for detoxification, without in-depth analysis of how family factors affect substance users, and then urge them to put an end to their drug use. At the same time, most studies still regarded family as an objective external factor, focused on what kind of family functioning/family support system/family relationship status could provide a good environment for substance users to put an end to their drug use. Although some studies have suggested that the effect of family factors on the motivation of detoxification were limited by the psychological state and individual cognition of substance users, they still focused on the influencing factors, and they have not revealed how individuals perceived their family and how this perception and understanding could promote the enhancement of detoxification motivation. These are questions to be answered in this study.

## 3. Materials and Methods

### 3.1. Research Design

The present study aims to explore how family factors facilitate the substance users’ motivation for detoxification. It is a complex phenomenon and varies among individuals within the family, social and cultural context. Qualitative research emphasises the holistic understanding of events or behaviours within the context, and enables the researchers to view the participants holistically within their natural settings [80]. Through qualitative research, the process of enhancing the substance users’ motivation to stop using drugs can be understood in detail within their family and broader social context. After learning about what types of problems are best suited for each approach to qualitative inquiry, the researchers determined to use a phenomenological approach in this study. The focus of this study was to understand several drug rehabilitants’ common experiences of enhancing their motivation for detoxification, in order to develop practices or policies. This is the typical type of problem best suited for phenomenological approach [81]. In detail, the researchers expected to obtain a deeper understanding from the drug rehabilitants about their experience with drug detoxification, how do they interpret these experiences, and what contexts or situations affect their motivation for drug detoxification. This is also consistent with the focus of phenomenological approach, which explores the lived experience of individuals and how individuals make sense of their experiential world [82]. Therefore, the qualitative research method and phenomenological approach was adopted in this study.

### 3.2. Participants

In this study, the researchers adopted criterion sampling strategy to look for participants who have shared an experience, but vary in characteristics and in their individual experiences [83]. The objective of this research was to explore how to enhance the motivation for drug detoxification, thus the criterion for sample selection in this study was that participants had the experiences of substance use and success in ending their drug use. Under the introduction of the leader of the drug rehabilitation group, the researchers approached 41 potential participants among the drug rehabilitants of drug treatment social work service projects in Shanghai, China. This kind of introduction by a key informant with high reputation laid the foundation for the smooth entrance of researchers, and also provided great help for researchers to gain the trust of interviewees in the process of conducting in-depth interviews and helping them communicate with researchers sincerely and frankly. Among the 41 possible participants, the researchers selected informative participants who were closely aligned with the research objectives. Additionally, the researchers recruited participants varying in rehabilitation years and demographic characteristics such as gender and age. During the process, the researchers checked the availability of enough in-depth data showing the patterns, categories and variety of drug detoxification experiences. The researchers jointly decided whether the sample size was sufficient. When data saturation was reached, the researchers stopped recruiting new participants. In total, 15 drug rehabilitants were finally selected as the research sample. The basic information about the 15 participants is shown in Table 1, in which the first letter of the numbered subjects is the interview time order, and the second letter is the gender.

### 3.3. Data Collection

In this study, face-to-face in-depth interviews were used as the main method, supplemented by participatory observation to collect data. Two researchers conducted in-depth interviews in Chinese with 15 drug rehabilitants in Shanghai, China, from August to September 2017. In order to obtain the richest information, the researchers conducted in-depth interview with each participant for three to four hours. Although it required a long time, the researchers used some methods to reduce participants’ fatigue. The researchers and the participants reached an agreement on the interview place near the participants’ residence or workplace, and preferably chose a setting devoid of loud noises, such as a private room of the cafe or tearoom, in which the participants might feel comfortable and relaxed. Before the interview, the researchers informed the participants beforehand about the time that would be taken for completing the interview and that the participants would have the right to take a break or stop the interview when they felt tired. Semistructured interviews were conducted via the use of an interview schedule comprising questions regarding demographic information, their personal life story, drug rehabilitation experience and their motivation to change. All interviews were recorded with the consent of the interviewees. In fact, the participants had strong willingness to share their experiences, and they cherished such communication opportunities since they expected that their rehabilitation experience could be seen by more people through the researchers’ writing, so as to influence more people to change their views on drug rehabilitation groups. Therefore, during the whole interview process, they all showed great energy. At the end of the interview, they even lamented on how quickly time passed.

For those in particular who had been recovering for a long time, they had difficulty providing more detailed information about the specific circumstances and practical feelings of their early drug rehabilitation experience. The long gap in time was more likely to lead to recall bias or affect their judgement in their description of past experiences. In terms of mutual verification of data, the researchers collected the documents such as the life story written by participants during participation in the drug treatment social work service project or articles about their experiences of drug rehabilitation written by participants that were published in related WeChat official accounts. To a certain extent, it was helpful to avoid the deviation of research conclusions caused by missing information provided by the participants. In addition, the researchers also participated in various drug treatment social work service activities. Through participatory observation, the researchers listened and watched the drug rehabilitants in mutual contact, and had the opportunity to communicate with more drug rehabilitants, so as to obtain a direct sensory impression of them and their explanation of the meaning of their words and deeds.

### 3.4. Data Analysis

Verbatim transcripts were typed according to the interview recordings and were then translated from Chinese into English by the first and fourth authors of this research. Finally, the second and third authors double-checked that the meanings of the translated transcripts in English were loyal to the original Chinese meanings, so as to ensure the highest level of accuracy. Qualitative analysis began with sorting and becoming familiar with data by the first and fourth authors. Two researchers read the transcripts two to three times, grasped the data and information as a whole, and marked the sentences that could describe the participants’ rehabilitation experiences and evoke strong emotions. Two researchers then analysed the key ideas of the transcripts, field notes and memos, and performed open coding separately based on actual data [84]. Two researchers often discussed the coding during the analysis process. Crosschecking of transcript coding was carried out by the second and third authors. Based on a careful examination of the data, the researchers developed as many codes as possible, such as “filial piety”, “parental responsibility” and “family reputation”. Points of divergence in coding were discussed until the research team reached agreement. Then, the researchers made a coding sheet to collect the codes and clustered them in preliminary categories. According to the preliminary analysis, the researchers interpreted that motivation of drug detoxification was strongly influenced by the cultural norms related to family intergenerational responsibility. This interpretation was based on a certain interpretative framework, which aimed to reveal the concepts behind the story that support the experience of research objects [85]. After repeated online and offline discussion, four categories were identified by the research team: compensation of intergenerational deficit, sharing of intergenerational reputation, constraint of role norms and expectation of intergenerational care. In the next step, the researchers made a further analysis based on the connotation of ethics at the value level and its guidance to the individual practice level, and divided the similar categories into two themes. The former two categories were summarised as the value aspects of family ethics, which refer to the influence of family ethics on the consciousness of the individual and thus shapes their values. The latter two categories were sorted out as practical aspects of family ethics, which refers to the influence of family ethics on the actions of individuals and thus guide their choices of action. Each theme was named with content-characteristic words, including “value orientation and consciousness stimulation of family intergenerational ethics” and “practical orientation and behavioural constraints of family intergenerational ethics”.

Four strategies [86] were adopted to enhance the trustworthiness of this study. (1) Prolonged engagement—the researchers spent a long time building a trust relationship with the participants by participating in their activities, which helped the researchers become familiar with them and clarify or elaborate any unclear points by going back to talk to them. (2) Data triangulation—the combinations of in-depth interviews, documentation collection and participant observation helped the researchers to approach the “reality” from different dimensions. (3) Member check—the researchers also repeatedly communicated with participants about their feelings concerning the interviews and the preliminary analysis findings, and discussed some of the research conclusions with them in the later stage. Thus, the researchers could find and reflect on the deviation in the process of data analysis and interpretation, and to some extent, it also ensured the validity of this study. (4) Peer debriefing—the researchers often held discussions together, as well as with the substance use treatment workers. Their comments, critiques and suggestions stimulated the researchers to reflect on the research, and further improved its credibility.

### 3.5. Ethical Considerations

This study was reviewed and approved by the Institutional Review Board of the first author’s PhD school. Informed consent had to be obtained from all participants to ensure they had the right to participate in the study. Therefore, when the interviewees were invited by the leader of the drug rehabilitation group, the purpose and methods of the research and the content that might be involved were initially explained by the principal researcher, and the independent will of the participants was emphasised. After the voluntary participants were identified, a written “informed consent form”, including the research background, research purpose, data collection methods and measures to protect the privacy of the interviewees, was sent to them through the WeChat platform, to confirm again whether they were clear about participating in the study. Finally, before the formal face-to-face interviews began, the interviewees’ understanding of the research content and confidentiality rules was once again confirmed orally.

In accordance with the norms of research ethics, the information about the participants was presented anonymously to avoid inadvertently exposing the identity of the participants. In this study, although the participants often appeared in public places, such as in anti-drug publicity activities, media interviews and public welfare services, at the same time, some participants also made it clear in the interview process that they did not mind their identity being disclosed, but rather expected to guide more people to stay away from drugs through their own experiences and to advocate a better community acceptance atmosphere through their positive change after rehabilitation. However, to ensure anonymity and security, the real names of research participants were not used in the study, and the recordings were not labelled with names. Instead, they were marked with numbers and destroyed after the study.

## 4. Results

In China, under the influence of Confucian family-oriented thought, people are mainly positioned in the basic pattern of human relations order, such as kinship and extended social relations [87]. Therefore, the responsibilities and obligations undertaken by individuals and the fulfilment and enjoyment of their rights are be constrained by family ethics. In contrast to Western family ethics, which focuses on the horizontal relationship between husband and wife, the Chinese family structure focuses on the vertical parent-child relationship [88], which emphasises filial duty whereby parents should be respected and children should be raised. Human ethics with the basic attribute of the “father–child axis” [89] has created a normative atmosphere among different generations of family members by clearly stipulating the parent–child code of conduct and the relationship between their roles and obligations [90]. The original motivation of the participants in this study to decide to put an end to their drug use superficially seemed to be some accidental events/situations, such as self-reflection after entering a compulsory detoxification centre for drug rehabilitation, family members’ effort to help or a word said to them, etc. However, what lies behind these seemingly accidental events/situations is the power of social norms of family intergenerational ethics; it rekindles their sense of intergenerational responsibility, which has been concealed by their craving for drug consumption, and then they are willing to make changes. In the words of the interviewees, these events or situations “poke straight into the soul”, “touch the softest part of the heart” and “open up the numbed good will within”. This paper discusses the enhancement of substance users’ motivation from the value orientation and the practical orientation of family intergenerational ethics.

### 4.1. Value Orientation and Consciousness Stimulation of Family Intergenerational Ethics

The value orientation of family intergenerational ethics reflects the basic principles of family order and the basic conditions for family to be placed in the whole society. Within the family, the relationship between power and duty stipulated by intergenerational ethics has become the guarantee of the orderly operation of the family, and the failure to assume the corresponding role obligations may cause damage to other family members. As for outside of the family, the family will be judged by its societal environment, which can affect the whole family’s image and honour, and it is the responsibility of family members to maintain them. Thus, when rehabilitants feel the potential or actual negative consequences of their failure to take responsibility for their families, they may realize that it is necessary for them to change their behaviour.

#### 4.1.1. Responsibility Assumption from the Perspective of Debt Compensation

When family members fail to perform their corresponding duties or even cause harm to the people they take care of, the psychological mechanism of debt will take effect. In this case, the anxiety and pressure perceived by substance users may prompt them to compensate by taking new actions or resuming their responsibilities. Compensation for harm caused by drug use in the past mainly occurs in the liability debt as parents or children. The former is more common, which may be related to the general belief that parents are duty-bound to raise their children to the best of their abilities.

As parents, they are naturally responsible for raising their young children, which is necessary for the continuation of humanity. Therefore, parents’ care for their children and offspring is rarely regarded as a strict moral requirement, but as something that everyone can do naturally, as part of the nature and instinct of parenthood [91]. For parents, “worrying” about their children and fulfilling their responsibility to raise them, which is to fulfil their cultural obligations, are often taken for granted [92]. Therefore, it is extremely rare that parents do not love their children at all, and so do the substance users. Although due to years of substance use, most of them have not accompanied their children as they have grown up, or their own past experiences negatively affected their children’s healthy development and future, their awareness of parenting and caring for their children did not disappear completely. Thus, when substance users are aware of the effects of their drug use experience on their children, they will feel deeply guilty about not taking responsibility for their children’s education and expect to make amends.

*I’ve been taken into the compulsory detoxification centre several times, never disciplined my daughter … The biggest responsibility for her mental problems is mine, because I did not assume my own responsibility. If I hadn’t taken drugs and had stayed with her, she wouldn’t be what she is today, would she? So, this is one of the major reasons why I don’t take drugs anymore, I must take my responsibility for the family, at least to take care of my daughter.*

*(NM)*

*If I didn’t get into (the compulsory detoxification centre) for two years, just stayed in the society, I couldn’t stop using drugs either, certainly I couldn’t. I do not know how many times I have tried to “quit” repeatedly, it’s useless! I have two sons. The older is over 30 years old and the younger one is only 15. When I got into the compulsory detoxification centre, my little son was not born yet. Just two days after I got in, he was born … At that moment, I thought, I’ve already ruined my elder son, I’ve never been with him, and I could not ruin the little son again. So, now at my age, all I need to do is to raise my little son well. That is, to take care of him and raise him, I don’t care about anything else.*

*(OM)*

In addition, when certain events or situations visually demonstrate to the substance users how their children have grown, their awareness of their indebtedness to their children and their responsibility in fulfilling their parental obligations will be stimulated, which in turn will arouse the expectation of being a competent parent again, thus becoming an important source of motivation for firm belief in drug rehabilitation. In this sense, the unfolding of a motivation to change is mostly initiated by watching one’s own children grow, and the role of parents will become increasingly more important as children grow older [93]. In addition, in some situations that may lead to relapse, the arousal of “parenthood”, and the roles and responsibilities that the identity places on the individual will also be a source of motivation to avoid relapse.

*The last day I came out of the female compulsory detoxification centre, there was one thing that touched the softest part of my heart, woke up my motherhood and reminded me of the responsibility that I should take as a mother. My daughter got her period for the second time on the day I came home. When I opened the door and walked into the bathroom, I saw a basin of my daughter’s underwear soaked in the water. The colour of the water made me feel a fierce pain. I entered the bedroom and saw the sheets with bloodstains that had not been replaced on the bed, and blood on the sofa, blood everywhere … At that moment, my tears would not stop, and I repeated a calling in my mind 10,000 times: “My baby, mum will never leave you again!” For the sake of my daughter, but also for myself, I will always stick to it, be a competent mother and no longer let my daughter grow up with any defects!*

*(Data collected from the article “Wake Up, it is Rebirth”, published in the Starfish column of the WeChat official account “Shanghai Anti-drug Volunteer Style”.)*

*I went through great family changes … I think I can withstand such a great change and deal with it well, because I put my position right. Because I was conscious at that time, and my responsibility was “I am a mother”. Now that I’m a mother, it’s clearer to me that my responsibility is not just to be myself, to be a normal self, but also to be responsible for my daughter. So that’s what keeps me strong in my belief that I will not look for any excuse to relapse.*

*(AF)*

The debt to parents is mainly reflected in the delay and compensation in the change of the family care role for substance users as grown children. In the Chinese society that emphasises filial piety, the patterns and directions of family care and support functions will change with the transition of the family life cycle in different stages. Generally speaking, young children grow older under the care of their parents, but when these children grow older and their parents become old, the role relationship of “care giver and cared” between the parents and children will start to reverse, and the children will take the role of providing care and support in the family [94]. In other words, children should repay their parents for bringing them up when they become adults, and become the givers of family financial support and life care, so as to ensure the quality of their parents’ life and help them enjoy their later years. Therefore, when substance users as the adult children in a family do not take the responsibilities of caring and supporting their elderly parents, but instead need their parents to take care of them or to worry about them, it can cause painful experiences such as shame and regret, so as to stimulate the awareness and motivation of change.

*The turning point for me was when my mother came to see me for the first time, which touched me the most. When she picked up the phone, she didn’t blame me for anything. Instead, she said, “Mom is not with you now. You should take care of yourself.” I just thought, I really look like a beast! The first feeling was that I was unfilial, I made a mistake, but I made my mother worry about me all day, which is a punishment for her as well, isn’t it? So, this is what urged me not to take drugs anymore, this is the biggest motivation and the original motivation!*

*(HM)*

At the same time, the awareness that parents may die will also have an impact on the lives of substance users, making them aware of their debt and responsibility to their parents. Studies have shown that these “worst of all” dilemmas often motivate substance users to reflect on themselves [95] and then be willing to start changing.

*The real break with drugs came two months after I got out of the compulsory detoxification centre. At that time, my mother earned a little money for me by weeding on the roadside. One day, a friend who worked in a hospital called me and said, “Did your mother go out to work today? Something went wrong and there are no survivors!” Later, my mother was fine. When she came home, she would sleep with me at night and hug me. One night she said to me, “Don’t take drugs anymore and stay at home. Anyway, if you have nothing to eat, Mom and Dad will give you food. If you have no clothes, we’ll buy for you.” After my mother said that, I really couldn’t stop crying, and then I was determined to “quit drugs”.*

*(BF)*

#### 4.1.2. Responsibility Assumption from the Perspective of Reputation Protection

“Home” occupies a prominent position in the Chinese culture [96]. To some extent, individuals are not the basic elements of society, but “home” is the core of society, and it is a “closely knit group” [97]. In this group, the individual’s sense of existence and value is not completely reflected in the self-realization of individual value, as life and death, honour and disgrace are primarily related to the interests of the whole family [98]. Thus, family members have obligations to each other, and share the honour and disgrace. In this sense, family responsibility is also reflected in not humiliating parents or having a negative impact on children’s future development through their words and deeds.

In terms of the identity and role of children in the family, “filial piety comes first in all virtues” is a long-term consensus in Chinese society. “Filial piety is the fundamental of Chinese society … We can see the influence of filial piety in all the activities of Chinese society and all lives of Chinese people” [99]. This influence is reflected in the physiological aspect of “body, hair and skin all come from parents and must not be damaged”, and the psychological aspect of “caring parents”, as well as the social aspect of “glorify the family” [100]. In other words, children repay their parents not only by serving them, but also by having more social meanings, namely inheriting their parents’ aspirations, achieving something and even glorifying the family. Therefore, in the process of children’s growth, they are expected to become brilliant and bring honour to the family; children should meet their parents’ requirements or expectations in a form that conforms to social norms. On the contrary, when children violate social norms, they not only consider the illegal aspect of individual behaviour, but also the negative impact of their behaviour on the family, resulting in a sense of guilt over their parents’ upbringing.

*A long time after I used drugs, I felt ashamed in front of my parents; they gave me life, no matter how, they raised me, and it didn’t seem like a good thing to do this … It was like doing … an impossible bad thing, the worst thing that could happen.*

*(DF)*

From this point of view, “filial piety” in a broader sense also includes all the norms of behaviour that conform to traditional ethics, so that parents will not be humiliated by them. As the old saying goes, “following virtue will be good for your relatives while doing evil will upset your family”. In other words, any behaviour that is not good or that worries parents is unfilial [101]. Under the influence of traditional filial piety, people even believe that it is evil for a person to break the law because it will bring harm to his/her parents’ personality or reputation [102]. Therefore, a person who is filial to his/her parents will never engage in illegal and criminal activities, because it is not conducive to filial piety to his/her parents, and makes his/her parents feel anxious and even discredits their reputation.

*I felt guilty when seeing my parents save me again and again and never give up on me. I also felt sad when seeing they still work hard. I felt that if they go on like this, they may experience the death of their child and be criticized by everyone. Behind them people will gossip that, “how is the son of this family?” I don’t want my family to deal with this kind of gossip.*

*(IM)*

Similarly, from the perspective of the parents’ role in the family, their responsibility of caring for children is not limited to financial support and life care, but also means that they should not discredit their children; that is, they should be responsible for their future. In China, under the influence of traditional concepts such as the “severe punishment doctrine”, the public generally show resistance and rejection toward criminals and even their families. Drug use is undoubtedly a violation of social systems and norms, and this behaviour will also cause serious harm to individuals, families and even the whole society, which makes it more difficult for drug users and their families to be accepted by the public, and they generally suffer discrimination and exclusion. At the same time, due to the implementation of political censorship in the recruitment process for specific positions such as joining the Party and civil servants in China, the illegal and criminal situation of immediate family members will have a certain impact on individual development. Therefore, in order to avoid the stigmatization effect in daily life or try to make up for the negative impact of the joint effect on their children in the formal system, substance users will solidify their belief in drug rehabilitation by taking their children’s reputation and future into consideration.

*After I went into the compulsory detoxification centre, it was my wife and my daughter who really gave me confidence to “quit drugs”. It was my wife’s words and deeds that saved my soul! She told me, “I care about you, and my daughter needs a complete family!” Ever since she talked to me, I’ve been thinking about it and even poked my soul! How could I face my daughter in the future? She’s definitely going to grow up. You can’t let her carry the heavy cross all the time, and be criticized by others that her dad is a substance user!” I’m so sorry for her! So I thought, “I can’t do this, I want to live again, don’t touch it!” Therefore, if it were not for the re-education this time, I may still be taking drugs, or I may already even be dead, right? Smoking to die! You were crazy out there! Crazy!!!! People may think of stealing wallets, stealing, robbing and so on; rich people like me, I was floating, floating all the time, there is an incredible feeling of floating up high all the time. When you get inside, you have time to settle down and reflect on the journey you experienced and everything in the past.*

*(KM)*

*When I was sorting things, I suddenly saw my daughter’s diary. I cried when I read one of them. The teacher asked her to write an application for joining the Party, she wrote, “How can I?” Three times, “How can I? How can I? How can I?” Three question marks, I know it was my fault and I felt very uncomfortable. Besides, she was looking for a job, and it seemed that the company had a good impression of her, but finally she didn’t take the job because of this. I have let my daughter down many times, so I can only be nice to her …*

*(FF)*

### 4.2. Practical Orientation and Behavioural Constraints of Family Intergenerational Ethics

As a kind of behaviour norm, family intergenerational ethics not only lie in preaching and introspection. People will also take this as a code of conduct, forming some kind of responsibility constraints [103]. In Chinese society, the influence of filial piety ethics on daily life practice is particularly prominent. It is emphasised that treating parents well and ensuring parents’ life is a responsible and binding duty for adult children [104], and most people in the society will fulfil this behaviour according to the convention [105]. Under the guidance and restraint of family responsibilities and obligations, substance users can reflect on their own role in the family, and then regulate their behaviour, before clarifying the direction of behavioural change.

#### 4.2.1. Responsibility Assumption from the Life Cycle Perspective

In contrast to the “relay mode” of the family life cycle in the West, the core feature of the family life cycle in China is a “repayment mode”, in which the next generation needs to undertake the obligation of supporting the previous generation in return for the kindness of their parents [106]. This means that the relationship between parents and children is a contractual relationship [107]; at different stages of family life cycle, parents and children meet their needs and take care of each other by raising young children and supporting elderly parents, respectively. According to the interview data, the situation of guiding substance users to take care of their families is mainly reflected in the later stage of the parent–child relationship when the parents are old and need to be taken care of, which may be related to the profound influence of the “filial piety” culture on Chinese people.

Under the influence of traditional filial piety and family values, the basic function of supporting the elderly in China is mainly undertaken by families, and the social expectation of providing for the elderly by adult children far exceeds the expectation of government welfare policies [108]. Even in the case of great changes in family structure and the heavy burden of family welfare, the concept of obligatory support for the elderly has always dominated [109]. In fact, this is closely related to Chinese people’s feelings and life values. It implies that the reason why everyone exists today is because our parents gave birth to us and raised us to grow up [110]. Naturally, children should take on the role of caring for their parents when they become old, and do their best to care for and serve them. In this regard, the traditional “filial piety” culture clearly stipulates how children should treat and serve their parents based on the ethical relationship within the family [111,112]. By defining family ethical responsibilities and obligations that they should assume as children’s identity and role, the filial piety of “caring for parents” can stimulate the conscience and ability of substance users and thus restrain and guide their behavioural change.

*My parents are old and need companion. Without a companion, their hopes for life may be shattered; at the very least, your companion is good for their health … It’s my duty to be there and accompany them, to make them healthier and happier.*

*(GM)*

*The last time I came back home and I saw a table full of dishes, and I said, “Is anyone visiting today?” My father said, “No, just waiting for you.” Later, my daughter told me that my father had prepared this meal for three days, and the seafood was bought at the wholesale market in the early morning. The other day I saw my father and I suddenly felt, how is he so old? I was thinking, taking care of my parents was my duty, but my daughter helped me. Am I going too far? So, for the next two or three years when my father was sick, I took care of him and actually repaid him.*

*(FF)*

On the other hand, from the perspective of the parent–child relationship and ethical obligations, in addition to “being born with courtesy”, the basic norms of “caring for parents” also include the fact that children needed to have their parents “buried and sacrificed with courtesy” after they died. Furthermore, the ideology and etiquette norms concerning this can be transformed into the concrete actions of people and have a real impact on them, which is mainly reflected in the concepts of “raising children as a guarantee against old age” and “serving the parents while living and arranging a proper burial after their death”. It is generally believed that whether children can provide and care for their parents’ retirement is an important aspect of their filial piety, and an important embodiment of whether parents could die a natural death. Therefore, children should fully serve and take care of their parents in their old age, and also repay their parents’ kindness by paying tribute and offering sacrifices after their parents’ death.

*I thought it all over when I was in the compulsory detoxification centre, and I told myself, I can’t go this way anymore, why? There is a Chinese saying, “serve the parents while living and arrange a proper burial after their death”. Nowadays Mum and Dad do not need your financial support, but you must arrange their funeral after their death. So I thought that when I came back and they didn’t pass away, I would never go this way again.*

*(EF)*

The expression “children want to serve but parents are no longer alive” aims to guide people to cherish the present opportunity of filial piety and take good care of their parents while they are alive. It can be seen that children’s caring for their parents is a kind of civilised behaviour, and there are usually different forms of “filial piety” instilled in children’s growth [113]. On this basis, personal emotions are actually transformed into normative emotions in roles [100], and children’s support for their parents is mainly based on social responsibilities stipulated by laws or relevant cultural traditions. In other words, children must support and honour their parents after they grow up, which is the moral responsibility and obligation they must assume as their children. Thus, the standard of filial behaviour and the role norms as children, which are perceived and gradually enhanced, will transcend the individual’s own attitudes or concepts [114], and form a basic value and cultural orientation for their daily behaviour and social relations [115]. As a result, when parents reach the age when they need to be taken care of, the identity role of substance users as the children in the contractual relationship and the corresponding responsibility norms will stimulate their motivation to change.

*My father came to visit me at the compulsory detoxification centre on crutches and told me that he missed me very much and was afraid he would never see me again. This kind of scene, which could usually only be seen on TV, actually happened in my life at that moment. For the first and only time in my life, I saw my father cry that day. After the interview, looking at my father’s trembling figure, the feeling of guilt for my parents was aroused spontaneously. In this world, there is a regret that cannot be made up for, that is, “the trees prefer calm but the wind will not stop, the children want to serve but the parents are no longer alive”. At that time, I prayed in my heart: don’t let this regret happen to me! At this moment, what I need to do is “quit drugs” and do my duty as a daughter.*

*(Data collected from the life story written by participant LF during her participation in the drug treatment social work service project)*

*What’s the biggest reason to “quit drugs”? My parents are quite old now and they always work hard for us. I feel they are too old, too old. This time if I go the same way again, maybe I will never see them again once I get inside.*

*(JM)*

#### 4.2.2. Responsibility Assumption under the Perspective of Intergenerational Mutual Assistance

In China, family provides the most fundamental welfare support and life security for individuals, and forms a virtuous cycle of intergenerational mutual assistance tradition between parents and children [116]. Even in current Chinese society, family mutual assistance is still widespread and its intensity even exceeds government assistance [117]. As a result, different generations form a community of shared interests, which can maintain the overall operation of the family through the flow of resources between generations. Thus, the obligations relationship between generations will take the principle of reciprocity into account, emphasizing that it is not only giving that is important, but also that what is given will be rewarded [118]. To sum up, giving has two meanings: one is supporting and caring for elderly parents as children; the other is nurturing and supporting young children as parents. The reward is more the expectation that their children will repay when they grow up, so that they can receive the same family care when they are old. In other words, important elements of intergenerational relationships also include expectations and demands formed within the family that individuals need to rely on the family to provide care and support for them.

However, some substance users do not form new families or raise children, which means that the family-centred intergenerational “repayment mode” in China cannot form a closed loop when they become old. In this case, their expectations of family care and support are more likely to fall on their parents. Even for families with siblings, once their parents die, the original family network will collapse [119] and there is no guarantee that they can obtain help and support from their siblings. At the same time, it is important to recognize that when substance users become old, their parents will have become even older, nearing their time of dying, or already have. As a result, substance users have to manage the situation of how to think about their future when their families are unable to care of them after the death of their parents.

*Why do I stop using drugs? My brother and sister are very kind to me and help me all the time. My mother is alive, my brother and sister would help me for the sake of my mother of course. But what if my mother is gone? Who will take care of me? In fact, I know, my mother is over 80 years old, she certainly will pass away, and then who will care about me after she has gone?*

*(CM)*

Specifically, as parents become aged or die, substance users will gradually realize that they can no longer rely entirely on their parents for their love and that others around them will not support them as much as their parents, so they can only bear all the consequences of their choices, decisions and actions on their own. In fact, we all know this very well in our daily life, that no matter how old we are, as long as our parents are alive, we can always be their children. When parents are about to leave or have already passed away, however, we can only face the future by ourselves. Without the family protection of intergenerational mutual assistance, substance users need to take corresponding moral responsibility for their own life and future, which will encourage them to realize “self-responsibility” through positive changes in behaviour.

*The last time I came out, my brother said to me, “Now the situation is getting tighter, you will enter the compulsory detoxification centre again if you are not careful, aren’t you tired? You’re 48 years old now. Can you do anything? Anyway, it’s your own life, if you want to go on like this, we can’t help you.” That is to say, if you go on like this, no one will support you anymore. We are all old, right? Mum’s gone, and you can only rely on yourself, right?*

*(MF)*

## 5. Discussion

This qualitative study explored how cultural and normative elements within the family affect substance users to enhance the motivation for drug detoxification. A total of fifteen rehabilitants were interviewed, seven of whom were female and eight were male. At the time of the interviews, the youngest participant was 35 years old and the oldest was 63 years old, with an average age of nearly 52 years old. Most of them have children (except CM, DF, GM, HM and LF) and many of the participants’ parents were older (e.g., AF, BF, CM, EF, FF, GM, HM, JM and LF) or deceased (e.g., MF) at the time when they made their determination to put an end to drug use. Analysis of the interview data revealed that the events/situations that motivated participants to develop strong motivation were related to their parents or children, and themes related to family intergenerational ethics began to emerge.

Among the fifteen participants, eight were motivated by family intergenerational ethics at the value level, which led them to realize the need to compensate for their missing family responsibilities through behavioural changes. There are two categories under this theme, namely, the compensation of intergenerational deficit and sharing of intergenerational reputation. Among them, five participants (AF, BF, HM, NM and OM) determined to put an end to their drug use because witnessing the growth of their children or their parents’ aging led them realize that they have owed to their family too much and decided to take family responsibility, and prompted them to reflect on their drug use behaviour. Three participants (DF, IM and KM) were motivated by the negative impact of their drug use on their parents’ or children’s reputations and began to come off substance use with this sense of guilt.

The motivation of the other seven participants for detoxification came from the correction of family intergenerational ethics at the practical level, which created constraints on how they should and could make changes and assume family responsibilities. This theme can also be divided into two categories: role norms constraint and intergenerational care expectation. Among them, five participants (EF, GM, JM, LF, FF) were guided by ethical norms related to intergenerational responsibility to initiate behavioural changes, two participants (CM and MF) chose to achieve self-care through putting an end to their drug use due to the constraints of family intergenerational mutual support norms.

### 5.1. Why Can Trigger Events/Situations Motivate Drug Rehabilitation?

Some researchers suggest that trigger events/situations are usually a kind of “hitting rock bottom” experience [120,121] or a “worst of all” dilemma [122], which can enhance the strong willpower of substance users [123]. We strongly agree that the behaviour change motivation of substance users is associated with the occurrence of major life events or life turning points. Moreover, although most of these events/situations are associated with various negative outcomes derived from drug use, they can still be a source of motivation for substance users to receive treatment [124]. Therefore, it is necessary to ask further why some specific events/situations can be the turning points for substance users to decide to change.

Previous studies suggested that the difficulty of turning points brought dramatic life changes that substance users had never experienced in their past life, and this difference and impact could help these substance users rethink the negative consequences of drug use [18]. Other studies suggested that compulsory supervision by the judicial system was also an important turning point in substance users’ life, as they provided sufficient time and space for self-reflection that led to active motivation and behaviour [125,126]. It can be seen that both the direct impact brought by the event itself and the turning point induced by the passive situation actually be emphasised that the internal thinking and reflection caused by the event/situation was more critical than the external event/situation itself. This ability of reflection and self-consciousness is crucial for substance users to fully rid themselves of their drug addiction. If they take action without deep reflection, they may use drugs again under the promotion of certain conditions [127]. In our study, the reason why triggering events/situations arouse participants’ deep reflection was related to the family ethics and intergenerational responsibility that are emphasised in Chinese society.

In fact, a number of studies found that substance users’ discontinuation of drug use was related to their family roles, particularly their new family roles and responsibilities [128], or their new understanding of those roles and responsibilities [129]. Some researchers also suggested that the role and responsibility of substance users in the family should be strengthened [130], and the special family ethical values in Chinese society should also be emphasised [131]. Consistent with previous findings that, although the development of the nuclearisation of family is one of the important characteristics of the family with the social transformation [132], institutionalized family roles and corresponding responsibilities have often been understood as traditional and underdeveloped in the past. Actually, however, the foundation of familial internal order and family culture has not been eliminated, and the family is still the moral source of virtue and has the religious function of securing one’s life [133]; this study found that, as a member of the family, an individual’s consciousness and action would inevitably be affected by the family. Therefore, the assumption of family roles and responsibilities needs to be examined back in the family order or family norms. Family order and family norms are collectively called “family ethics” by Liang Shuming, who defined Chinese society as an ethics-based family society [134]. Our findings indicated family ethics not only led to cognition and internalization at the value level, but also to constraints and norms at the practice level, which enabled substance users to eliminate the influence of elements that do not conform to the requirements of family ethics, and place the focus of life and the support of value on the responsibility within the family, so that they could make up their minds to put an end to their drug use and return to society.

### 5.2. Implications for Policy and Practice

The results of this study showed that in order to stimulate substance users’ intrinsic motivation to change, in addition to unilaterally intervening directly against the substance users themselves or their external family environment, it is necessary to bear in mind that substance users have their own initiative as a member of the family, and they have their own understanding and interpretation of the family under the influence of the family environment. It is important to help substance users realize the importance of family to them and guide them to appreciate their roles and responsibilities within the family. In this way, individuals and families can be truly connected, rather than separated from each other. Family is not just an objective external factor for substance users, but they are a part of the family and deeply influenced by it. This influence is not only reflected in the ideological level, but also internalized in action. Therefore, it is necessary to help substance users to see in what ways they are influenced by their families and how they themselves understand these influences.

Existing anti-drug laws and regulations in China, such as the Anti-Drug Law of the People’s Republic of China and the Drug Rehabilitation Regulations, mainly stipulate the duties and tasks of governments at all levels, as well as the public security organs, judicial administration departments and administrative departments for health, in assisting substance users to put an end to their drug use; however, the important role of family in drug rehabilitation has not been emphasised in related policies. Therefore, families need to be included in the implementation of various rehabilitation measures, such as voluntary drug rehabilitation, community-based rehabilitation and compulsory isolation. This means not only stipulating how families should participate in drug treatment as entities and their corresponding responsibilities, but also specifically promoting the education and publicity of traditional Chinese family culture in relevant drug rehabilitation facilities. For social workers providing drug rehabilitation services on the front line, it is necessary to recognize that in addition to the improvement of family relationships and the construction of a good family support system, it is also necessary to give attention to the significance of traditional culture in fostering individual values and behaviours, and cultivating individuals’ sense of responsibility and consciousness. For example, services such as writing life stories and taking photos of life can be adopted to guide substance users to think about their relationship with their families and their position in the family, or to design family education service projects with the theme of intergenerational family ethics. This research can provide directions for the services described above with the topics of ethical value-oriented family responsibility deficit and family reputation damage, ethical practice-oriented family role norms and family mutual assistance norms.

In addition, consistent with previous studies, this study found that significant events/situations and the resulting reflections are motivations for drug abuse behaviour change. Therefore, when providing social work services for substance users, social workers need to be highly sensitive and grasp the important turning points in the life of substance users, which can be used as the starting point for thinking about and discovering the possibility of change. At the same time, some similar simulation scenes can be established in the practical process, such as places to experience activities isolated from reality or role-play activities showing family members’ interaction, and creating “sitcoms” about family intergenerational responsibility, etc., which all aim to provide space and time for substance users to become calm and review their own lives and think about their future development. This is what substance users cannot do while using drugs, but it is essential for drug rehabilitation.

## 6. Conclusions

This study demonstrates the original motivation of 15 Chinese substance users to end their drug use and analyses how their motivation is guided and constrained by family intergenerational ethics.

**Figure 2:**
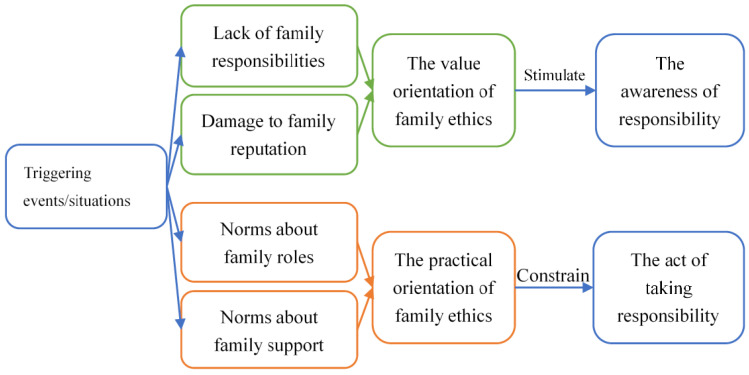
The mechanism of family ethics facilitating the motivation for drug rehabilitation.

The research found that certain events/situations triggered the internalized family ethics virtue of substance users, which implied the dual orientations of ethics in the family: On the one hand, when the events/situations made the individual perceive that they were in debt to their families for not taking the corresponding responsibilities during drug use, or their family members’ reputation and future were negatively affected because of their previous drug use behaviour, it would involve an external value judgement. In other words, based on the internalized ethical requirements and the recognition of their values, the rehabilitants would make judgements on their own behaviour, which would arouse a strong sense of reflection, thus stimulating the desire and motivation for behavioural change. On the other hand, when the events/situations prompted individuals to clarify their identity and role in the family life cycle, or when the family mutual assistance system failed to work normally, the responsible subject’s self-orientation would involve the ethical norms in specific practice scenarios, thus constraining themselves to make the corresponding behavioural changes in order to conform to the role orientation and responsibility norms of the parent–child intergenerational relationship in family ethics (see Figure 2).

Whether it is the guidance at the value level or the constraints at the practical level, these triggering events/situations ultimately lead to the assumption of family responsibilities in fact—that is, to realize that one needs to assume the family responsibilities or to clarify what kind of family responsibilities one should undertake. It is this commitment to family responsibility that enables them to find some external support from their family, and then leads to their own internal feelings about the need and courage required to fight against the suffering of drug addiction and other difficulties related to detoxification, thus stimulating the motivation for self-change.

This study explores how intergenerational family ethical contribute to the enhancement of motivation for drug rehabilitants in an ethic-oriented society such as China. Actually, this is still an exploration of the relationships among family members in a broader sense, only that the focus of this study on family relationships is different from previous studies. Therefore, this study enriches the discussion dimension on family relationships, and for some other countries and regions, it can also be used as a basis for further exploring the role of responsibility-taking among family members in promoting the motivation for drug rehabilitation. On the other hand, although the cultural paths of the West and China differ from each other, few countries and regions place as much emphasis on family ethics as China does, which extensively permeate into the relationship between individuals and society. However, family is not unique to the Chinese and the relationships and mutual care between family members of different generations is not a unique issue that only exists in Chinese society. In other words, in any culture, there must be responsibilities and obligations among family members, but varying in degrees. Thus, our study actually provides a reference for exploring how to promote the formation of drug detoxification motivation from the perspective of intergenerational responsibility-taking. Furthermore, as mentioned above, what matters is not these particular ethical norms and their specification of what and how family responsibilities should be assumed, but rather how substance users understand and interpret family ethical norms and the assumption of responsibility, and the consequent impact on individual behaviour. In this regard, the study reminds us to change the concepts and perspective whenever we carry out relevant research or provide drug treatment. That is to say, we should notice that substance users are not passive recipients of the influence of particular factors in the family, and we must see the agency of substance users in the process.

The current study has some limitations. First, most of the participants in this study were born between the mid-1950s and mid-1970s, and the type of drugs they used was predominantly opioid. Therefore, this research has not yet focused on the characteristics of substance users in the new period and the rehabilitation environment they faced, particularly the significance of the family to the individuals and its impact on their drug use behaviour change. Second, this study takes Shanghai, China, as the field site, and does not provide an in-depth comparative analysis of possible differences in family-influenced motivation for substance users in terms of geography. These are all relevant topics that can be pursued in the future, or some issues that need to be addressed when applying the results of this study.

## Figures and Tables

**Table 1:** Basic information about the participants.

Serial Number	Gender	Age (at the Time of Interview)	Level of Education	Type of Drug Used	Drug Use Severity	Use Fixed Number of Years	Years of Rehabilitation (at the Time of Interview)
AF	Female	43	Technical secondary school	opioids	dependency	11 years	13 years
BF	Female	42	Primary school	opioids	dependency	15 years	5 years
CM	Male	58	Junior high school	opioids	dependency	9 years	14 years
DF	Female	45	Technical secondary school	opioids	dependency	11 years	8 years
EF	Female	58	High school	opioids	dependency	10 years	14 years
FF	Female	57	Junior high school	multiple substances	dependency	24 years	8 years
GM	Male	54	High school	opioids	dependency	13 years	10 years
HM	Male	50	College	multiple substances	dependency	19 years	4 years
IM	Male	35	High school	opioids	dependency	6 years	12 years
JM	Male	57	Junior high school	opioids	dependency	14 years	6 years
KM	Male	56	High school	opioids	dependency	7 years	14 years
LF	Female	47	Technical secondary school	opioids	dependency	14 years	10 years
MF	Female	61	Junior high school	opioids	dependency	8 years	13 years
NM	Male	51	Junior high school	opioids	dependency	16 years	9 years
OM	Male	63	Junior high school	opioids	dependency	15 years	12 years

## Data Availability

Not applicable.
